# Effects of Advance Care Planning on End-of-Life Indicators for Nursing Home Residents—An Experimental Study with a Retrospective Chart Review

**DOI:** 10.3390/geriatrics9020042

**Published:** 2024-03-27

**Authors:** Yu-Tai Lo, Jin-Jy Wang, Yi-Ching Yang, Chiu-Yen Yu, Chia-Ming Chang, Ya-Ping Yang

**Affiliations:** 1 Department of Geriatrics and Gerontology, National Cheng Kung University Hospital, College of Medicine, National Cheng Kung University, Tainan 701, Taiwan; loyutai@outlook.com (Y.-T.L.); yiching@mail.ncku.edu.tw (Y.-C.Y.); 10108040@gs.ncku.edu.tw (C.-M.C.); 2 Department of Nursing, College of Medicine, National Cheng Kung University, Tainan 701, Taiwan; ns127@mail.ncku.edu.tw; 3 Department of Family Medicine, College of Medicine, National Cheng Kung University, Tainan 701, Taiwan; 4 Department of Gerontological and Long-Term Care Business, Fooyin University, Kaohsiung 831, Taiwan; zj825@fy.edu.tw; 5 Department of Medicine & Institute of Gerontology, College of Medicine, National Cheng Kung University, Tainan 701, Taiwan; 6 Department of Nursing, National Tainan Junior College of Nursing, Tainan 700, Taiwan

**Keywords:** advance care planning, advance directives, nursing homes, end-of-life care

## Abstract

Advance care planning (ACP) has the potential to improve the outcomes of end-of-life care for residents in nursing homes. The aim of this study was to determine whether an ACP program was beneficial for nursing home residents by assessing end-of-life indicators. An experimental study with a retrospective chart review was conducted. In total, 37 residents in the intervention group participated in an institutional advance care planning program for 1 year, and their chart data over 1 year were collected following the completion of the program; 33 residents in the control group had died within 1 year before the start date of program, and their chart data were reviewed retrospectively. Chi-square and *t* tests were used to examine four indicators of the quality of end-of-life care. Compared with the control group, the intervention group had a higher proportion of do-not-resuscitate directives, hospice care before death, and deaths in the nursing home, and fewer hospitalizations and deaths in an emergency department. ACP programs may improve the quality of end-of-life care for nursing home residents in Taiwan. Further research across different long-term care facilities is warranted.

## 1. Introduction

Nursing home residents have multiple comorbidities with a high incidence of emergency transfers and hospitalizations, and they rarely survive after attempted cardiopulmonary resuscitation or aggressive life-sustaining treatments. In addition, the majority of nursing home residents prefer to forego cardiopulmonary resuscitation at the end of life, which indicates the importance of advance care planning (ACP) for this demographic [1,2,3].

Although ACP guidelines have been implemented and various initiatives have been taken to increase the number of advance directives (ADs) signed in nursing homes, the methods and outcomes of ACP remain uncertain [4]. ACP is a process that supports patients by understanding and accommodating their personal values, life goals, and preferences regarding future medical care [5]. Studies conducted in Western countries have indicated that ACP interventions are beneficial for nursing home residents [6]. Specifically, these benefits include increased palliative care referrals [7], an increased completion rate of ADs [7,8], an increased number of residents dying in their nursing homes [9] and no-tube-feeding directives [10], and a reduction in end-of-life hospitalizations and medical costs [7,9,10]. Therefore, ACP is considered a vital component of end-of-life care for nursing home residents, and it has received policy and legislative support in Western countries such as the United States, the United Kingdom, and Australia since the 1990s [3,11]. In the United States, Australia, and Germany, 10–20% of adults have completed ACP [11]. However, as of 2021, fewer than 0.1% of adults in Taiwan have ACP registration [12].

Although end-of-life care is regarded as the most important component of ACP, discussing treatment goals and the completion of an AD are the major goal of institutional ACP programs [13]. A regulatory framework to ensure that nursing home residents are informed of their right to accept or deny written AD is needed [14]. Furthermore, in Asian countries, death is a taboo topic and many people consider signing an AD to be bad luck [14]. In Taiwanese nursing homes, the rate of signing do-not-resuscitate (DNR) directives is only 16.4% [15], in contrast to 40% in the United States and 75.8% in the United Kingdom [16,17]. In addition to the markedly low prevalence of DNR directives, superstitions regarding death also hinder the progress of early and ongoing ACP discussions and may lead to unnecessary hospitalization, hospital death, and ineffective treatment for nursing home residents as they near the end of their life [18]. Therefore, a national framework for ACP programs is urgently needed.

Despite its importance being acknowledged, ACP is challenging in Asia. Studies have documented the benefits of ACP programs for nursing home residents in Western countries, but few studies have researched the effect of ACP programs on the indicators of end-of-life care for nursing home residents in Asia [13]. Studies have demonstrated the positive effects of ACP programs [1,2,3], but cultural differences between Western and Eastern countries remain an obstacle. A narrative review of ACP in Asia revealed that ACP is difficult to initiate and conduct due to fear of conflict with family members, legal consequences, and the lack of a standardized system for ACP; moreover, patients are rarely invited to engage in the ACP process. The local contexts can set appropriate expectations of ACP outcomes and help establish the need, standardized across studies, to tailor interventions [13,19].

The present study documented the implementation of an institutional ACP program at a nursing home in Taiwan and evaluated the changes in the end-of-life care indicators of nursing home residents after the implementation of the program.

## 2. Methods

### 2.1. Study Design and Participants

We conducted an experimental study with a historical control at an urban, 200-bed accredited nursing home in Kaohsiung City, Taiwan. The study institution was government-certified, with an A+ quality rating. The nursing home is affiliated with a 225-bed community teaching hospital that provides counseling, home care services, and inpatient hospice care. Convenience sampling was used. All eligible participants in both groups were former residents of the study institution. A one-year ACP program was implemented. The intervention group comprised residents who had been receiving ACP program and died during the 1-year period after the one-year ACP program was implemented. Exclusion criteria included the residents who did not participate in the ACP program and died during this year and living residents. The control group comprised residents who died during the 1-year period prior to implementation of the nursing home’s ACP program who died without receiving ACP program service. If the resident or family members wished to discuss end-of-life care preferences, then the nursing staff referred them to physicians to discuss residents’ DNR directives. Both groups were analyzed through a retrospective chart review.

### 2.2. ACP Program

The purpose of the ACP program was to (1) assist nursing home residents and their families in understanding the concepts and principles of ACP, (2) promote awareness of residents’ and families’ autonomy over their lives, (3) assist residents and families in making healthcare decisions that align with their values at the end of life, (4) alleviate the physical and emotional burdens of residents and families at the end of life, (5) improve the quality of care for nursing home residents. A standardized program to meet local context should be promoted, and we invited two physicians from the Board of Hospice and Palliative Care to assist with the program standardization. First, we conducted three education and training sessions on ACP regarding the process and options of end-of-life healthcare decision-making, relevant laws, and procedures for advance directives, and communication skills training to promote effective communication for all nursing staff before program implementation. Second, we developed education material about ACP for residents and families. Third, we established a comprehensive resident health assessment mechanism every 3 months to regularly assess residents’ palliative care needs, including physical condition, cognitive function, psychological status, religious beliefs, and life values. All nurses involved in the ACP program received training in ACP discussion prior to the program’s implementation.

The program proceeded in three steps. First, prior to nursing home admission, the head nurse would arrange at least one meeting with the resident or their family members to discuss care preferences and goals for end-of-life care, and AD documents were included with a yearly contract and a form indicating consent to being admitted. At this stage, residents and their families frequently asked questions such as the following:

1. What does an AD include?
2. How do we sign an AD?
3. Can we modify the AD or other documents at any time?
4. Who can we consult if we have any questions or uncertainties?

The second step involved one of the physicians immediately initiating ACP discussions with the resident or their family members upon admission. Common questions at this stage included the following:

1. Do we need to initiate ACP discussion and sign the AD now?
2. Who should participate in the ACP discussion?
3. What topics will be covered in the discussion?
4. What if we need more time to consider our options?

The third step involved initiating conversations about ACP again when identifying a decline in the resident’s health. This decline could be indicated by frequent emergency department visits, hospitalizations, new diagnoses of severe diseases, or unexplained functional decline. During this stage, family members frequently asked questions such as the following:

1. What signs or symptoms might indicate a decline in the resident’s health?
2. Why is it important to discuss ACP at this stage?
3. What treatment options will be discussed?

Throughout the program, the two palliative care physicians were responsible for providing ACP consultations to all residents, as needed.

### 2.3. Ethics Approval

This study received ethics approval from the Institutional Review Board and was performed in accordance with the Declaration of Helsinki.

### 2.4. Data Collection

Demographic data, specifically those on sex, age, major diseases, length of institutional care, functional status, indicators of quality of end-of-life care, and place of death, were collected. Functional status was measured using the Barthel index, which ranges from 0 to 100 and depends on a person’s ability to perform self-care activities (personal hygiene, bathing, feeding, toileting, dressing, bowel control, and bladder control) and mobility in everyday life activities (stair climbing, ambulation, and chair/bed transfer). A lower score indicated greater functional impairment. The level of Barthel scores are interpreted as follows: scores of 0–20 indicate “total” dependency; scores of 21–60 indicate “severe” dependency; scores of 61–90 indicate “moderate” dependency [20]. The intraclass correlation coefficient and Cronbach’s alpha were 0.96 and 0.94, respectively. The criterion-related validity with Karnofsky Performance Scale and the functional capacity domain of the European Organization for Research in the Treatment of Cancer Questionnaire were 0.77 and 0.70, respectively [21].

### 2.5. Outcome Measurements

End-of-life care in nursing homes were assessed using four indicators: the increase in residents’ DNR directives and use of hospice services and the decrease in feeding tube using before death and number of hospitalizations during the final year of life [22].

### 2.6. Statistical Analysis

Continuous variables are presented as the mean and standard deviation, whereas categorical variables are presented as the frequency and percentage. The intervention and control groups were compared using the chi-square test or Fisher’s exact test for categorical variables and two-sample *t* test or the Mann–Whitney U test for continuous variables. Statistical significance was defined at *p* < 0.05. All tests were two-tailed. All statistical analyses were performed using SPSS Version 22 (IBM, Armonk, NY, USA).

## 3. Results

A total of 70 residents were included in the study; 33 were in the control group, and 37 were in the intervention group. These residents had a mean age of 84.7 ± 9.7 years (ranging from 46 to 102 years), and the mean length of institutional care was 1.9 ± 2.1 years (ranging from 0 to 5.1 years). The major underlying diseases were cerebrovascular diseases (38.6%), dementia (28.6%), cancer (25.7%), and diabetes (24.3%). The Barthel index scores for all participants ranged from 0 to 40, indicating total to severe functional dependency, although the intervention group exhibited a lower mean Barthel index score compared to the control group (1.8 ± 3.39 vs. 8.4 ± 7.76; *p* = 0.025), the number of participants with advanced dependency (total to severe dependency) was similar in both groups (*p* = 1.00) (Table 1).

In comparison with the control group, the intervention group had a higher proportion of residents who received hospice care prior to death (27.0% vs. 15.2%), had more DNR orders prior to death (83.8% vs. 72.7%), were more likely to use a feeding tube before death (89.2% vs. 81.8%), and had fewer hospitalizations during the last year of life (1.1% vs. 1.6%). The intervention group also had a higher proportion of residents die in the nursing home and the hospice ward (45.9% vs. 33.4%), whereas a lower proportion died in intensive care units and the emergency department (16.2% vs. 33.3%). However, no significant differences in the place of death were noted between the intervention and control groups (see Table 2).

## 4. Discussion

Although no significant differences between the intervention and control groups were found, the percentages of residents with a documented DNR order and receiving hospice care prior to death were higher in the intervention group [6,23,24]. These findings are inconsistent with those of previous studies [7,8]. In these cases, the surrogate participated in the ACP program and the resident relied on the surrogate to sign the AD documents. However, due to the influence of cultural factors, such as superstitions about death and Chinese familial roles, most surrogates were unwilling to sign the AD documents until the last moments of life [24,25]. In addition, in the control group, some family members of residents also discussed residents’ DNR directives and signed the AD documents. This may have obscured the effectiveness of the ACP program and highlights the importance of implementing ACP during the early stages of disease [1,24]. Another reason could be the quality and resources of the study institution. Because the nursing home in our study was certified by the government as having excellent quality of care, the medical staff may have already understood the concept of end-of-life care before the implementation of the ACP program. The study nursing home was also affiliated with a hospital, meaning that it had abundant palliative care resources. Nursing homes lacking material and human resources may encounter difficulties when implementing similar ACP programs. The unique characteristics of the nursing home in this study may have contributed to the effects of the program being statistically nonsignificant.

The feeding tube usage before death in the intervention group was higher than in the control group. However, no difference in feeding tube usage before death was discovered between the two groups [23]. The lower Barthel index scores with these two groups indicated great functional impairment, which may explain the high prevalence of feeding tube insertion in these two group. This finding is consistent with that of another study, demonstrating that even when the risks, benefits, and alternative options are explained, most families will opt for the installation of a feeding tube during end-of-life care [3]. Many families worry that residents may be starving to death due to their poor oral intake. Family members expect beneficial outcomes from feeding tube placement and view this intervention as a necessary measure to address feeding problems, increase patient comfort, and improve the quality of the end of life [10,26].

Hospitalization at the end of life is the most widely used indicator for ACP interventions in nursing homes, although varying definitions have been used and various results obtained [24]. We observed fewer hospitalizations and deaths in intensive care units and emergency department among patients who received the ACP intervention relative to the control group; however, the difference was nonsignificant [6,23]. Residents in these two groups had lower Barthel index scores, all of them have severe dependency, and their progressive, ultimately terminal illnesses are associated with poor responses to hospitalization [25,27]. The confounding factor of illness severity may have reduced the effectiveness of the ACP program. This finding is inconsistent with the results of the systematic review [9]. These studies reported a lower rate of hospitalization in patients who received an ACP intervention than in those who did not. This inconsistency can be attributed to the larger samples in the mentioned studies.

Developing mechanisms for discussing and recording residents’ preferences is crucial for improving the quality of end-of-life care in nursing homes [3]. Taiwan currently lacks a regulatory framework to ensure that nursing home residents are informed of their right to prepare a written AD. Hence, an institutional ACP program may improve the quality of end-of-life care for nursing home residents. A scoping review also recommended that materials such as videos and interactive multimedia be used to supplement facilitated discussions when educating residents and their families about ACP and that written ADs and clinician training have positive effects on the primary outcomes for end-of-life care [24]. The findings of this study may provide healthcare professionals and policy makers with the information needed to develop ACP programs and regulations tailored to the needs of older adult residents in long-term care facilities.

This study has several limitations. First, the control group was a historical control. Because we lacked a parallel control group, potential selection and reporting biases were unavoidable, and our results should be interpreted with caution. Second, the value of power for sample size is 0.72 using back calculation; the small sample may have affected the validity of our findings [28]. Third, due to the severity of illness of the residents, the majority of the active participants in the ACP program were family members, not the residents themselves. The existence of surrogates introduced a degree of bias, which may have obscured the effectiveness of the ACP program. Finally, the findings of our single-institutional study cannot necessarily be generalized to other nursing homes.

Most of the residents included in our study were severely disabled and therefore relied on family members to make end-of-life care decisions on their behalf. This highlights the importance of implementing ACP during the early stages of disease. In long-term care institutions, resident characteristics and facility resources are heterogeneous. Further investigation is warranted to better understand the benefits of ACP interventions for nursing home residents with various characteristics who are residing in facilities with varying levels of resources. In addition, the concurrence between treatment requested and treatment received should be considered to be one of the indicators, and a new prospective study using standard random control group methods is needed in the future [19].

## 5. Conclusions

Our study using comparative analysis demonstrated the effect of an institutional ACP program on indicators of end-of-life care at a nursing home in Asia. We anticipate that our study will raise awareness about the importance of ACP in nursing homes.

## Figures and Tables

**Table 1:** Comparison of baseline characteristics and outcomes in the intervention and control groups.

	Intervention Group (*n* = 37)	Control Group (*n* = 33)	*p*
**Baseline characteristics**			
Age (year) *	84.5 (10.5)	84.9 (8.7)	0.88
Number of comorbidities *	4.2 (1.3)	4.3 (1.4)	0.73
Female	20 (54.1)	16 (48.5)	0.64
Dementia	13 (35.1)	7 (21.2)	0.20
Cancer	7 (18.9)	11 (33.3)	0.17
Advanced Barthel index	37 (100)	33 (100)	1.00

Data are presented as frequency (percentage) or mean (standard deviation) *.

**Table 2:** Comparison of outcomes for the intervention and control groups.

	Intervention Group (*n* = 37)	Control Group (*n* = 33)	*p*
**Quality of end-of-life care, facility-level measures**			
Do-not-resuscitate directives before death	31 (83.8)	24 (72.7)	0.26
Hospice care before death	10 (27.0)	5 (15.2)	0.23
Feeding tubes before death	33 (89.2)	27 (81.8)	0.38
Number of hospitalizations during the last year of life *	1.9 (1.1)	2.5 (1.6)	0.09
**Place of death**			
Nursing home	10 (27.0)	6 (18.2)	0.38
Hospice ward	7 (18.9)	5 (15.2)	0.68
General ward	14 (37.8)	11 (33.3)	0.51
Intensive care unit	5 (13.5)	8 (24.2)	0.15
Emergency room	1 (2.7)	3 (9.1)	0.34

Data are presented as frequency (percentage) or mean (standard deviation) *.

## Data Availability

Data are available from the Institutional Data Access/Institutional Review Board of Antai Tian-Sheng Memorial Hospital (contact via tsmhirb2018@gmail.com) for researchers who meet the criteria for access to confidential data.
